# Ras Isoforms from Lab Benches to Lives—What Are We Missing and How Far Are We?

**DOI:** 10.3390/ijms22126508

**Published:** 2021-06-17

**Authors:** Arathi Nair, Katharina F. Kubatzky, Bhaskar Saha

**Affiliations:** 1 National Centre for Cell Science, Ganeshkhind, Pune 411007, India; nayerarathi@gmail.com; 2 Department of Infectious Diseases, Medical Microbiology and Hygiene, University Hospital Heidelberg, 69120 Heidelberg, Germany

**Keywords:** Ras, signaling, therapy, Ras-isoforms, H-Ras, K-Ras, N-Ras, oncogene, cancer

## Abstract

The central protein in the oncogenic circuitry is the Ras GTPase that has been under intense scrutiny for the last four decades. From its discovery as a viral oncogene and its non-oncogenic contribution to crucial cellular functioning, an elaborate genetic, structural, and functional map of Ras is being created for its therapeutic targeting. Despite decades of research, there still exist lacunae in our understanding of Ras. The complexity of the Ras functioning is further exemplified by the fact that the three canonical Ras genes encode for four protein isoforms (H-Ras, K-Ras4A, K-Ras4B, and N-Ras). Contrary to the initial assessment that the H-, K-, and N-Ras isoforms are functionally similar, emerging data are uncovering crucial differences between them. These Ras isoforms exhibit not only cell-type and context-dependent functions but also activator and effector specificities on activation by the same receptor. Preferential localization of H-, K-, and N-Ras in different microdomains of the plasma membrane and cellular organelles like Golgi, endoplasmic reticulum, mitochondria, and endosome adds a new dimension to isoform-specific signaling and diverse functions. Herein, we review isoform-specific properties of Ras GTPase and highlight the importance of considering these towards generating effective isoform-specific therapies in the future.

## 1. Introduction

**Figure 1:**
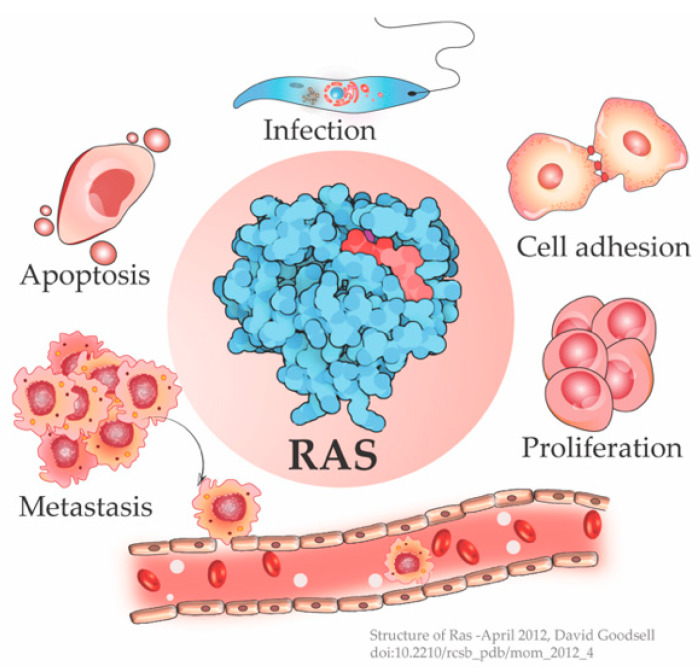
Ras is a signaling switch that regulates counteracting cellular functions like cell proliferation and apoptosis. Emerging studies have elucidated new functions of Ras e.g., in cytoskeletal rearrangement regulating cell adhesion and in parasitic infections like *Leishmania*, in addition to its well-known role in cancer progression. (Structure of Ras-April 2012, David Goodsell [5]).

The cellular Ras GTPase serves as a crucial molecular switch for non-oncogenic and oncogenic circuitry in cells. It is a tightly regulated key signaling molecule for many cellular processes as diverse as proliferation, cell adhesion, migration, differentiation, and death (Figure 1). Novel structural and functional properties of Ras GTPase in controlling tubulogenesis in endothelial cells [1] and pseudopodium dynamics in *Dictyostelium* [2] are emerging. Since the discovery of the roles of Ras in oncogenesis in the 1980s, the development of small molecular inhibitors against Ras has been the prime focus of pharmaceutical companies. Here, we review the structural and functional differences between the Ras isoforms and the outstanding issues including the druggability of Ras.

### 1.1. The History of Ras Isoforms—From Viral Oncogenes to Pivotal Cellular Genes

In the 1960s, Jennifer Harvey observed that the viral preparation from leukemic rat induced sarcomas in newborn rats. This oncogenic viral genetic element inducing Rat Sarcoma was named H-Ras (Harvey Ras) [3]. Later, while serially passing Mouse Erythroblastosis virus (MEV) in Wister-Furth (W/Fu) rats, Kirsten identified another retrovirus carrying the Ras gene [4]. Initially named as a variant of *Src* (sarcoma), this gene was not only mutated in a diverse spectrum of cancers but also encoded a 21k Da protein that had crucial cellular roles in normal cells.

**Figure 2:**
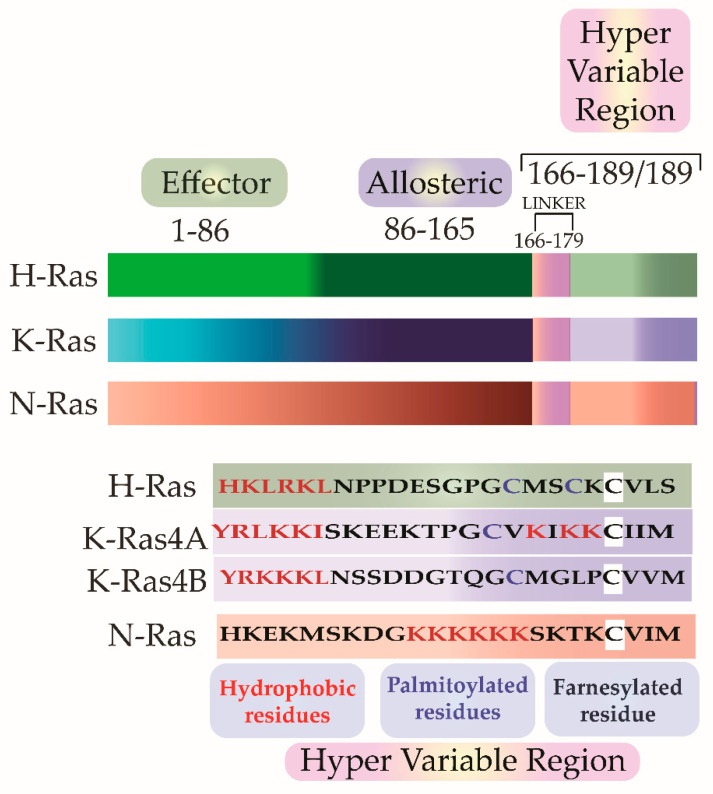
The isoforms share sequence similarities in their G-Domain (1–165 aa). This is followed by a Hyper Variable Region (HVR) (166–188/189), which differs in all three isoforms in sequence as well as in the post-translational modifications. In addition to farnesylation, a post-translational modification that all the three isoforms undergo, H-Ras and N-Ras further undergo dual and single palmitoylation, respectively. K-Ras undergo the addition of a stretch of polylysine residues.

Scolnick et al. first hypothesized and Stehelin et al. later experimentally proved that these oncogenes were the proto-oncogenes which, when mutated and virally transferred, were converted to transforming oncogenes [6,7]. As observed by Harvey and Kirsten, the retroviruses arose as a result of the passage of the murine leukemia virus through rats. The process of genetic recombination accounts for the presence of such cellular genetic elements in simple retroviruses. A plausible hypothesis, as framed by various researchers, holds that integration of a provirus may occur upstream of a cellular sequence forming a chimera of cellular-viral genetic elements. In subsequent replications, non-homologous recombination occurs between the cellular and viral genetic elements leading to the acquisition and incorporation of cellular genetic elements into the retroviruses [8,9,10]. Later in the 1980s, Scolnick and his colleagues found the cellular origin of this membrane-associated protein, which is dependent on guanosine-5′-triphosphate (GTP) binding for its activation [11,12,13]. It was discovered that three loci of this gene encode for four proteins with ~80% sequence similarity: Harvey Ras (H-Ras), K-Ras A and B (Kirsten-Ras), and neuroblastoma Ras (N-Ras). K-Ras gene encodes for two proteins K-Ras4A and K-Ras4B via alternative splicing. These splice variants have distinct membrane targeting sequences. Except for a few residues, these isoforms have identical amino acid (aa) sequences in their G-domain (aa 1–86), and variations lie in the allosteric lobe (aa 87–166) and the hypervariable region (HVR) (aa 166–178/179) [14]. The HVR can be further subdivided into a linker domain (aa 166–178/179) and a targeting domain, wherein the post-translationally modified residues lie (aa 179/180–189) [15]. As the fourth exon in K-Ras encodes for the HVR, there lie differences in the HVR of K-Ras4A and K-Ras4B. K-Ras4A undergoes palmitoylation, whereas, K-Ras4B, which lacks a palmitoylation site, adds polylysine residues (Figure 2).

### 1.2. Ras the Crucial Signal Relay Protein

The expression pattern of Ras isoforms is tissue-specific as well as developmental stage-specific [16,17]. The primary role of Ras emerged as a protein that assembles signaling complexes and relays signals to regulate an array of cellular activities. Emerging roles show its involvement in maintaining the integrity of actin cytoskeleton, in cell adhesion and migration, endocytosis, etc. [18]. This brings us to the crucial question—‘How does one signaling protein regulate counteracting processes like proliferation and apoptosis?’

Ras is a molecular switch that is activated by guanine nucleotide exchange factors (GEFs) that catalyze the exchange of guanosine diphosphate (GDP) with GTP. The counter process—the transition of Ras from active to the resting state—occurs by GTPase activating proteins (GAP) mediated GTP hydrolysis. Ras has a slow intrinsic GTP hydrolysis activity which is accelerated by GAPs by ∼10^5^ folds [19]. Two well-characterized GAPs are neurofibromin (NF1) and Ras P21 Protein Activator 1 (RASA1) (p120GAP) [20]. If Ras deactivation fails, Ras remains at an active or “On” state. Once active, Ras binds to a range of effectors that carry out the downstream signaling. The specificity, as well as the diversity of signaling, arises due to binding with specific activators and effectors [21]. Chin et al. and Fisher et al. demonstrated that Ras-induced oncogenic transformation by uncontrolled cell proliferation requires a sustained expression of activated Ras and in an inducible oncogenic system, withdrawal of Ras expression led to tumor regression [22,23]. The ability of Ras to induce both proapoptotic and antiapoptotic signals may depend on an interplay between the type of receptor(s) activated, strength of activation, the access to activators and effectors, their binding specificities, kinetics, and stoichiometry of signaling. The outcome of Ras activation depends on whether it is a normal or an oncogenic cell, as a highly activated Ras in a normal cell will most likely relay a proapoptotic signal, whereas in an oncogenic setup it will relay an antiapoptotic signal [24]. 

## 2. Ras in Cancers 

Ras is mutated in ~30% of cancers. Against the previous belief of p21 Ras as a single oncogenic entity, newer studies associated Ras isoforms with specific types of cancers. K-Ras mutations are the major contributors to most cancers. H-Ras mutation leads to cancers of the skin, head, and neck involving cutaneous squamous cells, whereas K-Ras is highly mutated in pancreatic, colorectal cancers, and adenocarcinoma of the lungs. N-Ras is frequently mutated in leukemias of myeloblastic cell type [25] and is implicated in myelodysplastic syndromes [26]. Oncogenic mutations frequently occur at residues 12, 13, 59, 61, and 63 impairing the GAPs mediated GTP hydrolysis to result in constitutively active Ras. As per the Cancer Genome Atlas (TCGA) project in 46% of cancers, the receptor tyrosine kinase–Ras pathway is altered [27]. Table 1 shows the percentage of point mutations in Ras isoforms in different primary tissues.

## 3. One Switch Many Outputs

Diverse signals like growth factors, hormones, cytokines, neurotransmitters, etc. can activate Ras. It acts as a common junction in the signal relay of many pathways. The most well-characterized pathway of Ras activation is through the growth factor receptors–Receptor tyrosine kinases (RTKs). This includes the epidermal growth factor receptor (EGFR), platelet-derived growth factor receptor (PDGFR), fibroblast growth factor receptor (FGFR), etc. Following growth factor stimulation, the RTKs undergo dimerization. The signal propagates downstream through the autophosphorylation of the adaptors like growth factor receptor-bound protein 2 (Grb2), which recruits GEFs like Son of Sevenless (SOS).

The Grb2/SOS complex binds to and activates Ras by facilitating the exchange of GTP for GDP. GTP-bound Ras undergoes a conformational change in its effector domains switch I and II, opening up more binding sites for effectors. Signals are further propagated through effectors of Ras containing the Ras-binding domain (RBD) like the serine/threonine kinases rapidly accelerated fibrosarcoma (Raf1) or phosphoinositide 3 kinase (PI3K) followed by a cascade of phosphorylation of MAPK modules [28]. Ral guanine nucleotide dissociation stimulator (RalGDS) is another effector of Ras which contains the RBD domain. It further acts as a GEF for the activation of Ral GTPases. Studies in skin cancer models have proven the indispensable role of RalGDS in Ras-induced oncogenesis [29]. 

The main MAPK cascades studied so far include extracellular signal-regulated kinases (ERK1/2), p38 mitogen-activated protein kinase (p38MAPK), c-Jun N-terminal kinase (JNK), and ERK5 [30]. One of the key signaling molecules activated by Ras–Raf1 is mitogen-activated protein kinase kinase ½ (MEK1/2) that further activates ERK1/2. MEK1 has a proline-rich sequence that is necessary for its binding to Raf [31].

The three lysines and other hydrophobic residues in the *N*-terminal part of MEK enable its binding to ERK1/2 [32]. Upon stimulation of ERK1/2, MEK translocates to the nucleus but is quickly sent back to the cytoplasm [33]. The intrinsic kinase activity of ERK is enhanced 1000-fold due to its phosphorylation by MEK1/2. ERK1 is phosphorylated at Thr202 and Tyr204 and ERK2 at Thr183 and Tyr185 [34,35]. ERK phosphorylates a wide spectrum of substrates in the cytoplasm, organelles, and nucleus [36]. Ras–Raf–ERK-mediated proliferative functions are mainly an outcome of the stimulation-induced translocation of ERK to the nucleus [37]. MEK induces phosphorylation of the threonine and tyrosine residues of ERK, exposing its SPS (SPS (Ser244, Pro245, and Ser246) motif, within the nuclear translocation signal (NTS) [38]. This enables binding with an escort protein importin 7 (Imp7) and thus the translocation of ERK to the nucleus. Once in the nucleus, Ras-related nuclear protein (Ran GTPase) dissociates Imp7 from ERK leading to the accumulation of ERK in the nucleus [39]. Accumulated phospho-ERK activates transcription factors like c-Myc and c-Fos which are key regulators of cell cycle progression [40] and proliferation [41], respectively. In addition, other transcription factors involved in chromatin remodeling and subsequent acceleration of the cell cycle like the upstream binding factor (UBF) are also activated by ERK [42].

p38MAPK transmits extracellular cues to intracellular targets and its signaling dictates cellular responses like proliferation, differentiation, response to stress [43], etc. A diverse range of MKK kinases activates p38 including TGF-β activating kinase (TAK1), Mitogen-activated protein kinase kinase kinase 4 (MEKK4), mitogen-activated protein kinase kinase kinase 5 (MAPKKK5), and dual leucine zipper–bearing kinase/mitogen-activated protein kinase upstream protein kinase/zipper protein kinase (DLK/MUK/ZPK). On activation by Ras, p38MAPK follows a similar translocation to the nucleus and regulates transcription factors. Ras by its association with interleukin-1 receptor-associated kinase (IRAK), TRAF6, and TGF-β activating kinase 1 (TAK1) is involved in interleukin 1 (IL-1) induced activation of p38MAPK [44]. p38MAPK binds to escort protein either Imp7 or Imp9 and Imp3 subsequently binds, forming an (Imp7/Imp3/kinase or Imp9/Imp3/kinase) complex. Similar to ERK translocation, Ran GTPase dissociates importin from p38MAPK [45]. An array of transcription factors are activated by p38MAPK which includes activating transcription factors (ATF 1/2/6) [46], Nuclear factor of activated T cells (NFAT) [47], erythroblast transformation specific transcription factor ELK1, serum responsive factor Sap1 (SRF) [48], C/EBP homologous protein (CHOP) growth arrest and DNA damage-inducible protein 153 (GADD153) [49], p53 [50], C/EBPβ, chromatin remodeling HMG box protein HBP1 [51], and various myocyte enhance factors like MEF2C, MEF2A [52], microphthalmia-associated transcription factor 1 (MITF1), DNA damage-inducible transcript 3 (DDIT3), etc. [53,54].

### To Divide or Not—Ras Decides the Cellular Fate

Ras plays a pivotal role in regulating the process of determining whether a cell should undergo proliferation or remain quiescent. Cell cycle progression depends mainly on cyclin-dependent kinases (CDK) comprising a kinase subunit that binds to a regulatory cyclin, forming a cyclin-CDK complex- an active kinase. A repertoire of other kinases and phosphatases is also involved in this process. In quiescent cells, Ras directs the progression of cells from G1 to the S phase by inducing the synthesis of DNA [55]. As mentioned earlier, transcription factors act as a substrate for Ras-activated ERK1/2 or P38MAPK. Lowy et al. showed that transient expression of oncogenic Ras in the cells leads to the expression of Jun and Fos that activate the transcription factor AP1 (Activator Protein 1) [56]. Retinoblastoma gene (Rb) plays a major role in the progression of cells from G1 to the S phase. In quiescent cells, Rb remains bound to the transcription factor EF2 (Elongation Factor 2) in a hypophosphorylated state. This in turn is bound to histone deacetylase HDAC1 that represses gene transcription [57]. Activation of CDK4/6 by cyclin D hyperphosphorylates Rb and it dissociates from the Rb-EF2-HDAC complex initiating the transcription of genes. Many studies have shown the Ras can induce the expression of cyclin D [58,59,60] by the Raf–MAPK pathway [61] and thus shorten the G-phase in cells. Ras upregulates cyclin A as well, which is important for Ras-induced anchorage-dependent growth [62]. A feeble expression of cyclin E is also induced by Ras [63], but the prime mechanism of progression of cells from G1 to S is by degradation of CDK inhibitor p27Kip1 by MAPK signaling [64]. Expression of Ras in rodent and human primary fibroblasts cells also induces cell cycle inhibitory p53 and p16INK4a (p16 Inhibitors of CDK4 a) which leads to cell cycle arrest [65]. In MCF10A cells, a human breast epithelial cell line, with constitutively active H- and N-Ras, the activation of MAPKs was elucidated. H-Ras and N-Ras both activated ERK1/2; however, neither isoform activated JNK1/2. p38MAPK was activated prominently by H-Ras and not N-Ras [66].

## 4. Four Decades of Research and Why Ras Remains Undruggable? 

Despite extensive research and understanding of Ras from the last four decades, Ras remains undruggable. The answer lies in the fact that the prerequisite for targeting a molecule for drug discovery requires an in-depth understanding of various nuances of its functioning. Initial studies and attempts to generate an inhibitor to block K-Ras undermined the isoform specificity. Owing to their similarity in structure, H-Ras was considered as the prototype of all the three isoforms, and the initial drug development process primarily focused on the structure of H-Ras. De Vos et al. first deciphered the X-ray crystallographic structure of human cH-Ras at a resolution of 2.7 Å. An intact protein of 189 residues, a catalytic domain of 1–171 residues with GTPase activity, catalytic domains activated at valine 12 and leucine 21, and their corresponding intact protein were crystalized [67]. Using various slowly hydrolyzing GTP analogs, Pai et al. further provided a more refined crystal structure of guanine nucleotide-binding domain of p21^Ha-ras^ in its active GTP-bound conformation [68]. Using computational analysis, binding pockets for small molecular inhibitor was sought after, but the problem arose as later discoveries pointed out the absence of deep hydrophobic pockets on the surface of K-Ras. As Ras, a cytosolic protein, undergoes a series of elaborate post-translational modifications (PTM), to be finally targeted to the inner leaflet of the plasma membrane or other compartments, the next approach to target Ras was to develop post-translational modification inhibitors. 

The C-terminal CAAX motif (C = cysteine, A = an aliphatic amino acid, and X = serine or methionine) first undergoes farnesylation or prenylation with the addition of a 15–carbon farnesyl isoprenoid to the cysteine of CAAX motif by the enzyme farnesyltransferase (FTase). This prenylated protein is inserted into the membrane of the Endoplasmic reticulum (ER). Here, the CAAX motif is cleaved, AAX removed by Ras converting endopeptidases (RCE1). The next step is carboxymethylation of the farnesylated cysteine by isoprenylcysteine carboxyl methyltransferase (ICMT). H-Ras and N-Ras undergo another addition of a secondary signal in the Golgi in the form of palmitoylation for its membrane targeting. Herein a 16–carbon palmitoyl chain is added to the cysteine upstream of the farnesylated carboxyterminal cysteine by a class of enzymes called palmitoyl acyltransferases (PAT). Palmitoylation occurs at cysteine 181 and 184 for H-Ras and at residue 181 for N-Ras, which makes these isoforms highly hydrophobic. Genetic studies in yeast have shown that Erf4p, a Ras modification peripheral membrane protein of ER, in complex with an integral membrane protein Erf2p, is required for palmitoylation as well as non-classical membrane targeting of Ras proteins [69]. 

**Figure 3:**
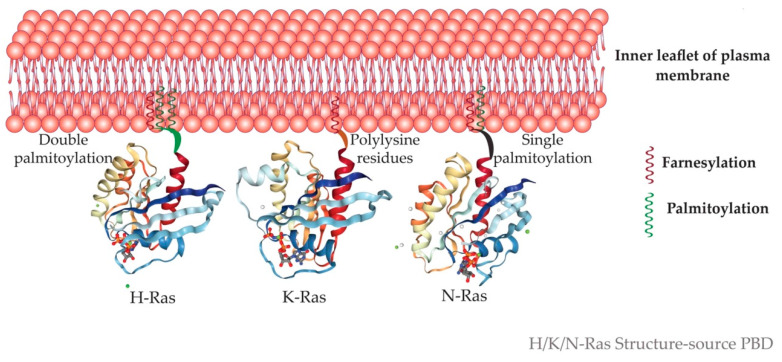
Ras isoforms are attached to the inner leaflet of the plasma membrane. Palmitoylation of residues 181 and 184 in H-Ras and residue 181 in N-Ras makes it hydrophobic and enables membrane attachment. The process of palmitoylation is reversible and depalmitoylated H/N-Ras rapidly shuttles between Golgi and Plasma membrane. Polylysine residues in K-Ras give it hydrophobicity and tether it to the plasma membrane. (PDB structures: H-Ras [73]; K-Ras [74]; N-Ras [75]; NGL (WebGL) viewer [76]. The membrane attachment structure is purely for visualization purposes only.

K-Ras follows a Golgi-independent targeting to the plasma membrane [70], where it is tethered to the cytoplasmic face. K-Ras is tethered to the negatively charged phospholipids of the inner leaflet by its polybasic lysine region, which is positively charged (Figure 3) [71]. Myristoylated alanine-rich C kinase substrate (MARCKS) is a protein that binds to the PM. Protein kinase C (PKC)-mediated phosphorylation of serine residues in the polybasic region causes neutralization of charges and hence MARCKS dissociates from the PM [72]. The association of Ras with the membrane is imperative to its functioning. Cyclic events of palmitoylation by PATs and depalmitoylation by acyl protein thioesterase 1 (APT1) lead to the redistribution of palmitoylatable Ras from the membrane back to the Golgi. 

Farnesylation is a process that all the three Ras isoforms undergo; hence farnesyl transferase inhibitors were branded as the next cancer drug. Peptide and non-peptide mimetics generated from the CAAX template that served as an alternate substrate for FTase, other thiols, and non-thiol FTIs were developed subsequently. Although FTIs such as lonafarnib, antroquinonol, tipifarnib, BMS-214662, and L778123 [77] showed promising results in preclinical trials, they failed in advanced phases of clinical trials. For example, FTIs showed no antitumor activity in pancreatic and colon cancers. Whyte et al. found that in the presence of a potent farnesyl transferase inhibitor, N-Ras and K-Ras proteins in human colon carcinoma cell line DLD-1 underwent an alternative prenylation using the enzyme geranylgeranyl transferase-1(GGTase) [78]. The next approach was to develop inhibitors for dual inhibition of both FTase and GGTase [79]. These inhibitors underwent clinical trials but failed due to their high toxicity and low specificity [80]. Since functional compensation of enzymatic activity is quite plausible, alternative use of statins was considered. This was based on the finding that farnesyl pyrophosphate is an intermediate in both cholesterol synthesis and prenylation. However, the rate constants for squalene synthase (an enzyme involved in cholesterol biosynthesis) and FTase are different, and hence the use of statins inhibited cholesterol biosynthesis to a higher fold when compared to farnesylation of Ras [81]. In addition, the therapeutic doses of Statin did not block the prenylation of Ras [82].

The CAAX processing enzymes and RCE1 and ICMT were lucrative targets for drug discovery. Various studies in mice showed that these two enzymes function in a broad and context-dependent manner. Deficiency of *Icmt* completely blocked transformation in rodent fibroblast, whereas *Rce1* did not have any pronounced effects [83]. In a K-RasG12D–driven oncogenic model of myeloproliferation, *Rce1* deficiency aggravated [84], while *Icmt* deficiency ameliorated the disease [85]. Contrary to this, in the same K-RasG12D-driven oncogenic model of pancreatic cancer, *Icmt* deficiency accelerated the disease [86]. Cardiac [87], and retinal toxicity [88] of Rce1 inhibition, and the lacunae that exist in our understanding of the mechanism of these enzymes further limit their use as potential drug targets. 

In addition to farnesylation, palmitoylation is another PTM that enables membrane targeting of H- and N-Ras. As mentioned earlier, palmitoylation increases the hydrophobicity of H/N-Ras and a cycle of palmitoylation and depalmitoylation also alters Ras’s subcellular distribution. Depalmitoylation removes Ras from the membrane and it localizes to the Golgi. Zinc finger DHHC (aspartate-histidine-histidine-cysteine) domain-containing protein 9 (DHHC9) and Golgi complex-associated protein 16 (GCP16) are Ras-specific PATs, that have garnered some interest. Chemical inhibitors like 2-bromopalmitate are extensively used in cell culture to block PATs, but their widespread toxicity limits their use. One study shows that specific inactivation of PAT DHHC9 reduces the leukemogenic potential of N-Ras in mice [89]. Thus, generating specific inhibitors against DHHC9 may seem like a promising approach to overcome the problem of toxicity. However, even in Zdhhc9KO mice, the palmitoylation of N-Ras was reduced but not abrogated suggesting a compensatory palmitoylation by another closely related PAT DHHC14. Acyl protein thioesterases (APTs) are responsible for depalmitoylating Ras isoforms. Using Protein Structure Similarity Clustering (PSSC), Dekker et al. developed the inhibitor palmitostatin B against APT1, which perturbated the deacylation cycle of H-Ras in Madin-Darby canine kidney (MDCK)-F3 cells (a Harvey murine sarcoma virus-transformed derivative of MDCK cell line). Inhibition of thioesterase activity over the long-term using Palmitostatin B leads to the loss of steady-state distribution of palmitoylated Ras. However, the study points out the fact that treatment with palmitostatin could also likely affect the spatial distribution of other palmitoylated proteins [90]. Moreover, these strategies would only be effective in H/N-Ras-driven oncogenesis and as K-Ras4B is not palmitoylated, would fail in about 85% of Ras-driven cancers caused due to K-Ras mutation [91]. As palmitoylation is a very dynamic process and its dynamics may vary in normal versus oncogenic cells, the generation of a palmitoylation/acylation inhibitor would require extensive research. 

This led researchers to design inhibitors against activators–guanine nucleotide exchange factors (GEFs) and effectors of Ras. GEFs and GAPs tightly regulate the activation of Ras isoforms by their association/dissociation to GTP. Son of sevenless (SOS) is one of the prime GEFs with multiple binding sites for Ras. Patgiri et al. developed a cell-permeable α-helix peptide that mimics the binding element of SOS, and hence the bound peptide was shown to inhibit SOS-induced activation of Ras and thus extracellular signal-regulated kinase-mitogen-activated protein kinase (ERK-MAPK) signaling in cells [92]. The two known members of the mammalian SOS family-SOS1 and SOS2 share about 70% sequence similarity and have similar expression patterns. As the SOS-induced activation of Ras is a tightly regulated process, mutations in SOS1 or SOS2 can lead to its enhanced activity and hence sustained Ras activation. SOS comprises six domains namely- (*N*-terminal histone fold (HF) or the H domain (HD), the Dbl homology domain (DH), the pleckstrin homology domain (PH), the helical linker (HL) domain, RAS-exchange motif (REM) domain, the Cdc25 domain, C-terminal proline-rich (PR) GRB2-binding (G) domain) [93]. In the inactivated state, the DH-PH domains allosterically autoinhibit SOS by occupying the Ras-binding site. This autoinhibition of SOS is released upon receptor-activated recruitment of SOS to the plasma membrane. Once recruited to the plasma membrane by PH, SOS is activated by binding of phosphatidic acid (PA) to the HD. Further, the REM anchors Ras-GDP and Cdc25 enables the dissociation of GDP [94]. A negative feedback loop controls the activity of SOS in Ras activation wherein SOS dissociates from its adaptor protein GRB2 upon phosphorylation of its C-terminal Serine/Threonine residues.

SOS1 is known to be functionally dominant over SOS2, as shown by knockout (KO) studies. Mice with constitutive knockout for *Sos1*, die in mid-gestation, whereas SOS2 is dispensable in mice for growth and development [95]. Moreover, functional redundancy exists between SOS1 and SOS2, as evident by the early death of SOS1/2 double knock-outs (DKO), whereas single KO of *Sos1*^−/−^ or *Sos2*^−/−^ are viable [96]. 

SOS mutations lie in either one or all of the above-mentioned six domains. One such rasopathy due to SOS mutation is Noonan syndrome (NS) characterized by developmental and cardiac defects. It is primarily an autosomal dominant disorder and based on the genes involved in the Ras signaling pathway that are mutated in NS, it is classified into 12 subtypes. As seen from Table 2 NS4 and NS9 are caused by mutations in SOS1 and SOS2, respectively. In NS4, caused by SOS1 mutation, around 183 mutations have been reported which affect 32 amino acid residues. These residues form various clusters—the first group comprises of {p.(protein) threonine 266 to lysine, p. methionine 269 to arginine/threonine, p.lysine 728 to isoleucine, p.tryptophan 729 to leucine, p.isoleucine 733 to phenylalanine} mutations in the DH and REM domains. 

The residues of this cluster are involved in regulatory autoinhibition (as mentioned above). The second functional cluster is in HF, DH, and PH domains (p.lysine 170 to glutamic acid, p.tyrosine 337 to cysteine, p.isoleucine 437 to threonine, p.cysteine 441 to tyrosine, p.serine 548 to arginine, p.leucine 550 to proline, p.arginine 552 to glycine/threonine/methionine/lysine/serine, p.leucine 554 to methionine 558 -deletion-insertion of lysine, p.methione 422 to valine, p.arginine 497 to glutamine, p.threonine 549 to lysine) [97]. The common missense mutations are p.arginine 552 to serine and p.arginine 552 to threonine) [98]. Arginine-552 is a crucial residue that interacts with aspartic acid 140 and 169 and any missense mutation in this residue hampers the interaction of HF and DH domain essentially affecting the autoinhibition of SOS1 [99]. The third cluster of residues (p.phenylalanine 623 to isoleucine, p.tyrosine 702 to histidine) are in the REM domain which interacts with Cdc25. The conserved residue phenylalanine-623 is crucial for the appropriate orientation and hence catalytic functioning of SOS1 [97]. Thus, these mutations primarily affect the residues that regulate the autoinhibition of SOS1. Hence such gain-of-function mutation of SOS1 keeps it on in its active state and hence the signal flow continues. The underlying effect of these mutations is enhancing the binding of SOS to the plasma membrane, which leads to sustained activation. This enhanced activation of SOS in turn leads to the hyperactivation of Ras.

Heterozygous mutations in SOS2 (chromosome 14q21) lead to NS9. All mutated residues (p.threonine 264 to lysine, p.methionine 267 to arginine, and p.threonine 376 to serine) lie in the DH domain. Mutations in these residues also hamper the autoinhibitory interaction of DH with REM and result in an unstable SOS2 in the state of rest. The GEF activity in cells expressing the above-mentioned mutations was higher. As this gain-of-function, the mutants showed higher activation of Ras and as well as enhanced MEK and ERK activation [100]. 

**Figure 4:**
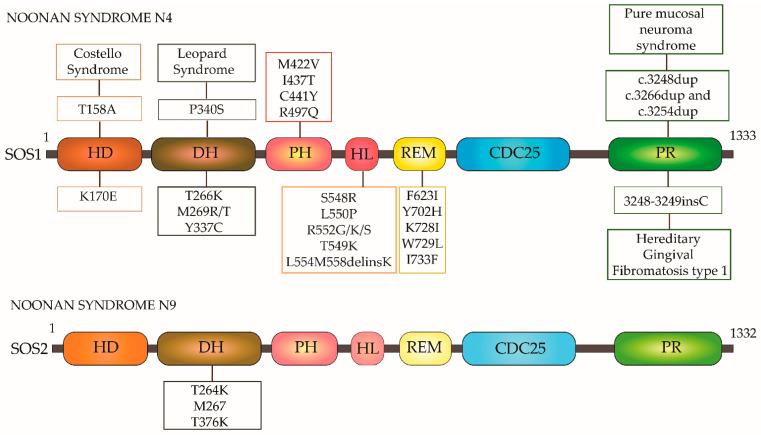
The various mutant residues of SOS1 In Noonan syndrome 4 (NS4), hereditary gingival fibromatosis type 1 (HGF-1), pure mucosal neuroma syndrome, Costello syndrome (CS), and Leopard syndrome (LPRD). SOS2 shares ~70% sequence similarity with SOS1 and mutation in its DH domain leads to Noonan syndrome 9 (NS9).

In addition to NS, an insertional mutation (ins) (3248_3249insC) induced frameshift results in a shorter SOS1 protein that lacks a proline-rich region (PR) involved in autoinhibition. Hence a constitutively active SOS1-Ras axis gives rise to hereditary gingival fibromatosis (HGF) [101]. In pure mucosal neuroma syndrome, characterized by mucosal neuromas, SOS1 is mutated as well. In addition to frameshift mutation c.3248dup(duplication); p. (Arg1084Lysfs*23) (arginine 1084 to lysine resulting in frameshift) [101], two more frameshift mutations (c.3266dup and c.3254dup) were identified that also result in gingival hypertrophy [102]. In Costello syndrome (CS) as well as Leopard syndrome (LPRD) SOS1 is mutated at HD and DH domains respectively [97]. SOS1 gene alterations are also identified in a spectrum of sporadic tumors. Methionine 269 to isoleucine/valine, glycine 434 to arginine, arginine 552 to serine/lysine/glycine/methionine, glutamic acid 846 to lysine, alanine 90 to valine/threonine, asparagine 233 to tyrosine/serine were identified in 1% of lung adenocarcinomas and uterine carcinomas [27] (Figure 4).

Other GEFs for Ras include Ras GRP, Ras GRF, Vav, etc. However, the activator specificity of Ras isoforms potentially poses a problem in the specificity of peptide inhibitors against GEFs and prompted studies towards targeting the effectors of Ras like phosphoinositide 3-kinase (PI3K). Mice with a germline mutation in the PI3KCα allele (mutation in Ras-binding domain) generated a variant of p110α which failed to get activated by Ras but still retained its non-Ras dependent functions. Cells from these mice showed defective growth factor signaling [103]. The limitation of targeting one effector of Ras like PI3K lies in the fact that Ras has multiple effectors like Raf1, adenosine di-phosphate (ADP) ribosylation factor (Arf), RalGDS, etc., and varying specificities under different contexts. 

## 5. Missing Pieces in the Ras Puzzle 

### 5.1. Multiple Receptors Activating the Same Relay Protein-Ras

Since its discovery as an oncogene, the prime focus of Ras research has been on its activation by RTKs. Most studies were unidimensional and undermined the isoform-specific signaling and functions. However, Ras is also activated by certain receptors primarily present on immune cells. Gulbins et al. in 1996 demonstrated that cluster of differentiation 40 (CD40), a tumor necrotic factor receptor (TNFR) family member, activated Ras. Treatment of Daudi B cells with anti-CD40 led to the activation of Ras which further correlated with the stimulation of Ras-related C3 botulinum toxin substrate 1 (Rac1), MEK-1, and PI3K [104]. This finding was further fortified by Nair et al., who showed that in murine macrophages, there exist activator and effector specificities for Ras isoforms wherein, a weak CD40 signal activated N-Ras via SOS, whereas a strong CD40 signal activated H-/K-Ras via Ras guanyl nucleotide-releasing protein (Ras-GRP). This signaling specificity of isoforms was further reflected in their specificity for effectors. H-/K-Ras showed specificity for PI3K at high strength of CD40 signal, meanwhile, N-Ras associated with Raf1 at a low strength of CD40 signaling [21]. 

**Figure 5:**
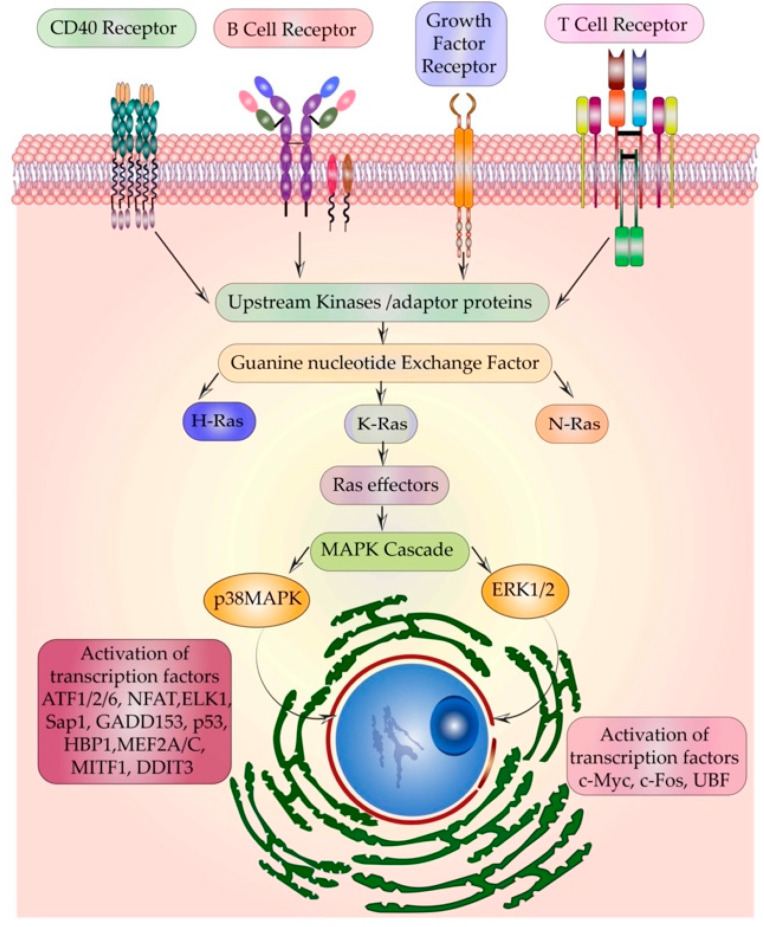
Ras is activated not only by receptor tyrosine kinases but also by receptors enriched on immune cells like B cell receptor, T cell receptor, CD40 receptor, and certain Toll-like receptors. On receptor ligation, the signal relay occurs through upstream kinases or adaptor proteins that activate guanine nucleotide exchange factors (GEFs). Ras isoforms have activator specificities that are context-dependent. Once activated H, K and N-Ras activate specific effectors that contain-Ras-binding domain (RBD). The signal is further relayed downstream via MAPK cascades which leads to activation of transcription factors and hence cellular responses.

Ras activation through stimulation of T cell receptors (TCRs) was first shown in human peripheral blood (Figure 5). Upon TCR stimulation p21 Ras was rapidly activated, as hypothesized then, due to a decrease in the activity of GAP on PKC stimulation [105]. Ras signaling plays a pivotal role in the early growth and maturation of T cells in the thymus [106] and the two main GEFs involved in the activation of Ras in T cells are SOS1 and RasGRP1. While SOS1 is required for the normal function of TCRs, a study in Jurkat T cells showed that following TCR stimulation, the activity of Ras GRP and GTP binding to Ras increased [107]. In addition, RTKs independently activate Ras through the Raf–MEK–ERK pathway to control dimeric transcription factor activator protein 1 (AP1) and NFAT. This leads to an increase in the expression of the IL-2 gene that drives T cell proliferation [108]. Not only TCRs but B cell receptors (BCR) activate Ras as well. On B cell receptor ligation, phosphorylation of its immunoreceptor tyrosine-based activation motifs (ITAMs) takes place which initiates signals to activate Ras via SOS. BCR stimulation causes the adaptor proteins Grb2 and Shc to form a ternary complex with SOS. Activated Ras recruits and activates Raf which further phosphorylates p21-activated kinases (PAK) and MEK1/2, which in turn activated ERK1/2 [109]. Ras GEFs control N-Ras activation by both individual actions and in concert. In both T and B cells, Ras-GRP is the dominant GEF for Ras activation and it also primes the Ras binding to the allosteric pocket of SOS. This creates a positive feedback loop that regulates the activity of Ras and provides a robust mechanism of activation of Ras even if a few lymphocyte receptors are engaged [110].

Not only the receptors of the adaptive immune system, but those of the innate immune system also activate Ras. Pattern recognition receptors (PRRs) like TLRs are found to activate Ras isoforms. Yang et al. found that upon stimulation of TLR 2, 3, and 4, by their agonists Pam_3_CSK_4,_ poly (I:C), and lipopolysaccharide (LPS) respectively, the expression of the PTM enzyme ICMT and Ras increased in macrophages. Similar effects were seen in the colon, stomach, and liver of mice with colitis, gastritis, and hepatitis. These results suggest that Ras plays an important role in TLR signaling that activates AP-1 mediated inflammatory response [111].

Another study reported that in macrophages, TLR9 ligand CpG ODN activates Ras in a dose-dependent manner. Following activation, Ras associates with TLR9 and promotes IRAK1/TRAF6 complex formation, leading to the activation of MAPK and NF–κB [112]. Ras isoform-specific activation by TLR was also observed, as Pam_3_CSK_4_ a TLR2 ligand increased the expression of N-Ras, but reduced that of K-Ras [113].

### 5.2. Ras Isoform-Specific Roles in Embryonic Development 

The differential involvement of Ras isoforms in development is also of prime importance. K-Ras is indispensable for embryonic development, as demonstrated by the embryonic lethality of K-Ras^−/−^. H-Ras and N-Ras are unable to functionally compensate for the effects caused due to the knockout of K-Ras in mice [114]. Nakamura et al. later found that there is a partial overlap in the functioning of Ras isoforms. *N-Ras*^−/−^/*K-Ras*^+/*−*^, died neonatally whereas, when compared to *K-Ras*^−/−^, *H-Ras*^−/−^/*K-Ras*^−/−^ embryos died much earlier during fetal development. Transgene expression of Human H-Ras in various mice mutants including triple mutant rescued the mice from lethality [115]. K-Ras4A and K-Ras4B are differentially expressed in mouse embryogenesis and in adult tissues. Although K-Ras4A is expressed early in embryogenesis, both inbred and crossbred mice with heterozygous deletion of exon 4A developed into healthy and fertile adult mice. This shows that K-Ras4A is dispensable for embryo development and the embryonical lethality of complete K-Ras knockout (lacking both splice variants) is due to lack of expression of K-Ras4B [116]. Another experiment showed the importance of H and N-Ras in pulmonary development in C57Bl/6 mice. Although the double knockouts (DKO) mice for H/N-Ras were viable, the number of DKO animals obtained upon breeding H-Ras knockout (*H-Ras*^−/−^:*N-Ras*^+/−^) with N-Ras knockout (*H-Ras*^+/*−*^; *N-Ras*^−/−^) were low in number. The underlying reason for the fatality of DKO offspring immediately after birth was pulmonary distress by the accumulation of ceramide in the lungs. This exemplifies the role of H-Ras and N-Ras in neonatal pulmonary maturation, which cannot be compensated for by K-Ras [117]. 

### 5.3. Ras and Infection

The research on the involvement of Ras isoforms in diseases other than cancers is still in its initial stages. Chakraborty et al. showed that H-/K- and N-Ras are differentially involved in *Leishmania major* infection. On infection, the expression of H- and N-Ras increases, whereas that of K-Ras decreases. Upon infection, the activation of N-Ras increased but that of both H- and K-Ras decreased. Higher expression but lower activation of H-Ras were correlated to its retainment in the Golgi possibly due to *Leishmania*-induced alteration of palmitoylation. N-Ras silencing using lentivirally expressed short hairpin RNA or a peptide (designed from the interface of SOS–N-Ras interaction) reduced *Leishmania* infection [113].

The role of Ras isoforms in antigen-specific immune response was also investigated. Using T-dependent and T-independent hapten-carrier conjugates, it was shown that silencing of N-Ras resulted in reduced carrier-specific T cells, reduced IL-4, and increased IFNγ production, whereas reciprocal effects were obtaining on H-/K-Ras silencing. Corroborating with this, N-Ras elicited a TH1 response, whereas H-/K-Ras gave a TH2 response. H-/K-Ras overexpression induced fewer Treg cells in Ovalbumin primed BALB/c mice when compared to N-Ras overexpression. These findings suggest that Ras isoforms can regulate antigen-specific immune responses in T cells [118].

### 5.4. Isoforms Yet Non-Identical–Subtle Structural Difference in-Ras Isoforms—The G-Domain Isn’t Identical

Except for the first 85 amino acids, the rest of the residues in Ras isoforms show only 80% similarity. Owing to these differences, only K-Ras undergo PKC mediated phosphorylation [119]. In addition to the sequence dissimilarities in the HVR region and differential post-translational modification in Ras isoforms, there exists a difference in the G–domain and allosteric lobe too. Emerging studies conducted using wild-type and Ras isoforms bound to GTP analogs have shown that even the G-domain in Ras isoforms are different [120]. There are 17 residues in the G-domain that is different for the isoforms. These differences lie in helix3/loop8 and helix4, between conserved ^116^NKCD^119^ and ^143^ETSAK^147^ motifs. Helix 3/loop 7 motif is crucial for connecting the active site of Ras to membrane-interacting residues. Additionally, residue 94 and 95 are different for all three isoforms. Position 95 is occupied by glutamine in H-Ras, histidine in K-Ras, and leucine in N-Ras. All these differences may lead to significant differences in the biochemical properties of Ras isoforms as well as in their signaling output.

The allosteric lobe interacts with the effector lobe and there lie isoform-specific differences in the *N*-terminal end of helix3/loop7, loop 8 preceding helix4, and C-terminus of helix 5 [121]. These isoform-specific differences can alter the interaction of the allosteric lobe with the active site of Ras isoforms. Sequence changes in Helix3/loop7 that link the active site to the allosteric site can affect the conformation [122], whereas sequence changes in Helix5 that contain nucleotide-sensor residues R161 and R164 linked to the active site can affect water-mediated hydrogen bonds [123]. Also, the residues near the nucleotide-binding pocket that may dictate the conformational transition of the active site are different in the isoforms. H-Ras has Tyr-141 whereas K and N-Ras have Phe-141. Such differences in residues can impact the configuration of the salt bridge that links the active-site motifs [124]. Isoform-specific residues A/P121, S/T127 for H-Ras, and Y/F141 lie around the salt bridge and may alter the way each isoform interacts in its GTP form. The proximity of these motifs to the residue pocket of Ras may also affect its interaction with the membrane [121]. One study has pointed out that the global conformational exchange of K-Ras-GppNHp is very similar to that of H-Ras [125]. Nair et al. reported that another parameter i.e., the local/global pattern of surface roughness of Ras isoforms is different. These differences can be mapped to functional differences between proteins as well as can dictate differences in protein-protein interactions [126]. The symmetry in functional site roughness of H/K-Ras is Surface_FD_ = 2.39, whereas that of N-Ras is Surface_FD_ = 2.25, which is significantly different [21].

### 5.5. Microdomain Localization of Ras Isoforms 

The prime platform of Ras signaling is the inner leaflet of the plasma membrane. The three isoforms localize to the cholesterol-rich-detergent-resistant raft, as well as non-raft depending on their state of activation. H-Ras is tethered to the plasma membrane by its HVR comprising of two palmitoyl residues. Studies using fluorescence-based vectors for H-Ras have shown that the targeting motif interacts with the cholesterol-rich domain. Another crucial motif in H-Ras’s interaction with the plasma membrane is the linker region that connects the effector domain to the HVR region [127]. On GTP loading, this linker induces a conformational change in H-Ras, laterally segregating it from a cholesterol-dependent to cholesterol-independent domain. Of the dual palmitoyl residues, Cysteine 184 is sufficient for the GTP binding dependent lateral segregation of H-Ras between the two domains. Contrary to this, if singly palmitoylated on cysteine 181, lateral segregation of H-Ras on GTP binding occurs from cholesterol-independent to cholesterol-dependent domains [128,129]. Galectin-1 acts as a scaffold protein to further stabilize the interaction of active H-Ras with the cholesterol-independent microdomain [130]. A few studies have pointed out that H-Ras itself might be important for raft formation, as it induces negative changes in the curvature of the plasma membrane [131]. This leads to the recruitment of more lipids into this area of curvature and thus a lipid-dense region is formed. Contrary to H-Ras, N-Ras undergo lateral segregation into cholesterol-dependent nanoclusters upon GTP-binding. In both H– and N-Ras, the lateral segregation is also dependent on the spacing between the palmitoyl group and the prenyl group [128,129]. K-Ras has a farnesylated cysteine and a polylysine residue for its interaction with the membrane. The important property of the polylysine residue is its negative charge which enables membrane tethering [132]. K-Ras resides in cholesterol-independent microdomains enriched with lipids like phosphatidylserine and phosphatidylinositol 4,5-bisphosphate. No study has yet elucidated GTP-dependent lateral segregation of K-Ras. However, galectin 3 binds to K-Ras-GTP and stabilizes it [133]. Latrunculin, an inhibitor of actin cytoskeleton completely abolishes the formation of cholesterol-dependent nanoclusters at the inner leaflet demonstrating the importance of intact actin cytoskeleton in this process [128,129].

Plowman et al. have also discussed in their study the relative size difference of Ras isoform nanoclusters in the inner leaflet of the plasma membrane. Using immunogold labeling of the plasma membrane microdomains, they found that the mean number of gold particles in the H-Ras G12V cluster was 2.5 that corresponds to about 6 H-Ras proteins, whereas for K-Ras G12V clusters it was 3.25, corresponding to roughly 7.7 K-Ras proteins. This difference in the size of the nanoclusters may translate into differences in the mechanism of nanocluster formation of the two isoforms [129].

A recent study showed the spatiotemporal preference of Ras isoforms at the plasma membrane [134]. Phosphatidylserine (PtdSer) affords K-Ras a capability to mechanosense membrane curvature and oncogenic K-RasG12V mutant prefers a flatter plasma membrane with low curvature. Corroborating with it, the K-Ras-dependent MAPK pathways are more active in flatter cells with low plasma membrane curvature [134]. Contrarily, dually palmitoylated H-Ras favor a curved membrane as also demonstrated by the finding that H-Ras–PI3K signaling is more stimulated in cells with a curved surface. Prostate cells expressing active H-Ras have an elongated morphology when compared to cells expressing H-Ras dominant-negative mutant H-RasT17N which have a flat morphology [135,136].

### 5.6. One Ras and Many Locations 

#### 5.6.1. Ras in Golgi 

The plasma membrane is the hotspot for Ras signaling; however, Ras is also present in the Golgi, Endoplasmic reticulum, mitochondria, and endosomes. Do these organelles also have a distinct pool of Ras activators and effectors? Growth factor-induced delayed and sustained activation of Golgi resident Ras has been visualized through fluorescent studies [137]. Moreover, the presence of Ras GEFs, GAPs Scaffold proteins (regulates the kinetics of signaling) Sef, and a Ras effector Rain1 [138] have been discovered in Golgi. Different GEFs are involved in the activation of Ras in this organelle. For instance, the activation of Ras on the Golgi is mediated by calcium/diacylglycerol dependent Ras guanyl release protein 1 (Ras GRP) [139], whereas in the ER, the activation of H-Ras is by Ras-specific guanine nucleotide-releasing factor 1 (Ras GRF1) and factor 2 [140]. Receptor endocytosis post-stimulation and interaction of the tail of the receptor with the cytosol account for the activation of Ras isoforms in endosomes. There also operates a pathway independent of receptor endocytosis that activates Ras in the Golgi through Src family kinases and phospholipase C-γ (PLC–γ). By the generation of second messengers required for the activation of Ras GRP, Ras activation occurs [141]. The activation kinetics of Ras in the Golgi is delayed but sustained unlike on plasma membrane which is rapid and transient. H-Ras, as well as N-Ras, are activated in Golgi. As mentioned earlier, palmitoylation of H and N-Ras affords hydrophobicity to it for its membrane attachment. A dynamic equilibrium exists between acylated and deacylated H-/N-Ras between the plasma membrane and the Golgi complex [129]. As mentioned earlier, two enzymes that take part in maintaining this equilibrium are Golgi resident palmitoyl transferase and acyl protein thioesterase. One proposed mechanism of receptor-induced activation of H-/N-Ras in Golgi is that on activation of these isoforms, APTs depalmitoylates Ras and hence mislocalizes it from the plasma membrane. This depalmitoylated H-/N-Ras reaches the Golgi again to be re–palmitoylated, contributing to the pool of active H-/N-Ras in the Golgi. N-Ras as it is singly palmitoylated has faster recycling and kinetics of activation when compared to H-Ras which has dual palmitoylation. Blocking the process of palmitoylation using inhibitors like 2-bromopalmitate, abrogated the pool of active Ras in the Golgi [142]. Raf kinase trapping to Golgi (RKTG), a Raf binding protein, and Sef both localize to Golgi. RKTG sequesters Raf in the Golgi in an inactive conformation, whereas Sef retains ERK in the cytosol [143].

#### 5.6.2. Ras and Endoplasmic Reticulum

The Endoplasmic reticulum forms another platform for Ras activation. Ras GRF, a GEF, is present in the ER. Immunofluorescence studies show that Ras GRF1 and RasGRF2 and SOS localize to the ER but not to the Golgi and activate H-Ras [140]. As mentioned in the earlier section, RasGRP mainly activated Ras in the Golgi. A transmembrane signal that retains Ras in the ER, activates MAPK cascade through Raf1 and leads to transformation. In addition to ERK, JNK and AKT are also activated by endomembrane Ras [140]. The kinetics of activation of Ras in Golgi and ER is more or less similar [70]. Signaling through Golgi and the ER regulates Ras signaling through MAPK scaffold. Wu et al. found that in response to oxidative stress and subsequent accumulation of misfolded protein, K-Ras in the ER stimulates unfolded protein response (UPR) and autophagy in human umbilical vein endothelial cells (HUVECs) [144].

#### 5.6.3. Ras in the Endosome 

RTKs and GPCRs activate Ras and both these receptors are endocytosed. During the process of endocytosis, the tail of the receptor is exposed to the cytosol and it can still signal. Guglielmo et al. showed that on stimulation of EGFR in the liver parenchyma, there was an internalization of the receptor followed by recruitment of Grb2-SOS within the endosomes [145]. This together with the finding that in rat liver early-sorting endosomes contain activated Raf1-MEK, a possible model was proposed. According to this model, upon EGFR stimulation, the dissociation of the Ras–Raf complex could take place from caveolae in the plasma membrane to the endocytic compartment [146]. Using the bimolecular fluorescence complementation technique Tsutsumi et al. visualized Ras activation in the endosomes. They observed the recruitment of Ras along with PI3K-RBD into the endosomes, which results in the accumulation of phosphatidylinositol 3,4,5-triphosphate (PIP3) [147]. Lu et al. also showed that on EGFR stimulation, K-Ras accumulates on EEA1/Rab5 bearing early endosomes, Rab7-marked endosomes, LAMP1/2- marked lysosomes, and multivesicular bodies [148]. Another study shows that on activation of TLR3 and TLR4 signaling in macrophages, RasGEF1b, a GEF is expressed in early endosomes. RasGEF1b binds to the GEF binding site of Ras that is required for the activation of the Ras–Raf–MAPK cascade [149]. Thus, the discovery of Ras GEFs and activation of its effectors in endosomal compartments further stratified signaling platforms for Ras.

#### 5.6.4. Ras and Mitochondria 

Mitochondria and the nucleus have also been identified to contain Ras isoforms. H-Ras splice variant which lacks the HVR region is present in the cytosol as well as the nucleus. The presence of N and K-Ras in mitochondria has been characterized. Cellular N and K-Ras were both found to be constitutively associated with purified mitochondrial fractions. N-Ras was detected in mitochondria purified from cultured cells and is associated with both the outer and inner mitochondrial membrane. While K-Ras is associated with the outer mitochondrial membrane [150]. N-Ras knockout cells showed abnormal mitochondrial morphology with concentric cristae. Normal morphology is reinstated in these cells by targeting N-Ras to the inner and outer mitochondrial membrane. K-Ras knockouts also exhibited similar mitochondrial structures that were reversed on ectopic expression of K-Ras in these cells [151]. A study by Rebollo et al. in 1999 showed the dependence of the expression of Ras isoform in mitochondria on IL-2 supplementation. K-Ras is present in mitochondria of IL-2 supplemented cells and its expression was not detected 12 h after deprivation of IL-2. Contrary to this, H-Ras was present only in mitochondria of IL-2 depleted cells. N-Ras was detected in mitochondria of both IL-2 depleted and enriched cells. Co-immunoprecipitation showed the association of K and N-Ras with Bcl2 in IL-2 supplemented cells and with H-Ras in IL-2 deprived cells [152]. This study also showed that the association of Ras isoforms is independent of their posttranslational modifications. 

**Figure 6:**
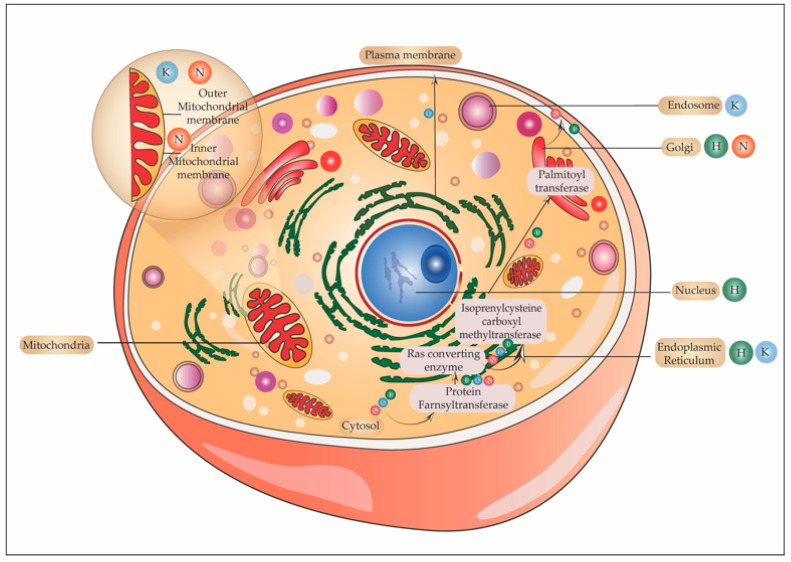
Ras isoforms are distributed and signals from not only the plasma membrane but also from other signaling platforms like Golgi, endoplasmic membrane, mitochondria, endosome, etc. Once produced in the cytosol, Ras undergoes a series of post-translational modifications in the endoplasmic reticulum and Golgi. Ras is farnesylated by protein farnesyl transferase (PFT), further proteolytically cleaved by Ras converting enzyme (RCE), and carboxymethylated by isoprenylcysteine carboxyl methyltransferase (ICMT). H/N-Ras enters the Golgi to be palmitoylated and subsequently targeted to the plasma membrane. while K-Ras goes addition of lysine residues and takes a Golgi-independent route to the plasma membrane. The presence of different Ras isoforms has been detected from the mitochondria, E.R, Golgi, endosome, and the nucleus.

Contente et al. reported the presence of H-Ras in both cytoplasmic and nuclear extracts, and treatment with FTI caused the loss of H-Ras from the nucleus. This presence of H-Ras in the nucleus synchronized with different phases of the cell cycle in both transformed as well as Ras-transformed and non-transformed cells. The peak of the H-Ras signal corresponded to the S-phase of the cell cycle implying a role of this isoform in replication [153]. Figure 6 shows the various subcellular locations of H, K, and N-Ras. 

### 5.7. Differential Access and Preference to Activators and Effectors 

The functional specificity of Ras isoforms can also be attributed to the differential access to or affinity of the isoforms for activators and effectors. This specificity is cell-type and activating receptor-dependent in most cases. Yan et al. elucidated the comparative activation of effectors PI3K and Raf1 by Ras isoforms: PI3K was activated to a higher extent by H-Ras when compared to K-Ras, which activated Raf1 [154]. The affinity of GAP NF1 is four-fold higher for H-Ras when compared to N-Ras [155]. Different groups have published similar cell types and context-dependent isoform-specific preferential activation of an effector [21]. Functional specificity of Ras isoforms may arise from:

- Different activation kinetics of Ras isoforms under the same receptor;
- Spatiotemporal segregation of signaling complex, with activators and effectors having different space, time, and thresholds of activation;
- Specific enrichment of substrates for activators or effectors in the microdomains containing specific Ras isoform.

For example, the enrichment of domain-containing any of the Ras isoforms with phosphatidylinositol 4,5-bisphosphate (PIP2) may increase its affinity for PI3K [156], whereas a phosphatidic acid-rich microdomain resident Ras isoform would preferentially activate more of Raf 1 [157].

## 6. Future Recommendations for Devising Isoform-Specific Targeting 

Various attempts have been made in the last four decades to target Ras but most approaches have seen no success. Functional compensation of enzymes is one of the main reasons for the failure of enzymatic targets. For example, geranyl geranyl transferase, functionally compensating for inhibition of protein farnesyl transferases (PFTs). This is exemplified by the functional overlap between Ras isoforms in some specific contexts. Similar approaches to target other enzymes involved in the post-translational machinery of Ras also didn’t prove to be promising. It clearly shows our lack of understanding of the complexity of structural and functional specificities of Ras isoforms. The key reason being that the functional diversity of the isoforms was overlooked in all such cases where a general inhibitor was sought after. 

K-Ras is most frequently mutated at codons 12, 13, and 61, which either perturbs its intrinsic GTPase activity or interferes with GAP-mediated GTP hydrolysis. However, the mechanism of such perturbations is different on mutations at different codons. K-Ras is mutated in about 90% of pancreatic cancers and 98% of these mutations occur at codon 12. The underlying basis of activating mutation at residue 12 is owing to the replacement of glycine with any other amino acid (except for proline), which leads to steric hindrance with the arginine of GAP [158,159]. The most common missense mutations are G12A, G12C, G12D, G12F, G12L, G12R, G12S, G12V [160]. Thus, mutation at codon 12 directly affects the binding and hence the activity of GAP. Biochemical and structural studies have further pin-pointed differences in the G12 mutant in terms of intrinsic GTP hydrolysis and preference in effector protein binding [161,162]. In G12C mutants, the cysteine residue is close to the nucleotide-binding pocket as well as the switch region that binds to effector proteins [163]. Small molecule inhibitors that covalently bind to this cysteine can effectively target G12C mutants. ARS-853 [164] and subsequently an improvised ARS-1620 [165] were developed which showed G12C mutant specific inhibitory activity. Since these inhibitors specifically interact with the thiol group of cysteine, the use is limited to G12C mutants. Currently, GTP analogs that can compete for the binding site of Ras are being developed like the one to target the G12C mutant. These analogs bind near the GTP-binding site or active site or between switch II and helix3. The limitation of these analogs lies in their inability to target the side chain of G12C mutant (the side-chains for mutation at positions 12, 13, and 61 are different) [166]. Another limitation of the K-Ras G12C inhibitor is that amplification of H/N or K-Ras levels in the cell itself can lead to dimerization and hence elevated GTP-bound Ras and thus confer resistance against the inhibitor. Another major challenge that lies in the way of generating a blocking inhibitor is the structural constraints in the structure of Ras isoforms. The structure of Ras is flat and it lacks binding pockets for such inhibitors.

Currently, only G12C is targetable because of the presence of a nucleophilic thiol of the cysteine group which can be targeted using covalently binding cysteine-reactive small molecules. Ostrem et al. identified this new allosteric pocket called S-IIP, which when occupied by a small molecule inhibitor shifts the nucleotide affinity of K-Ras from GTP to GDP state and also reduces its interaction with effectors and regulators [163]. 

In 2020, Amgen (Thousand Oaks, CA, USA) and Mirati Therapeutics (San Diego, CA, USA) introduced two K-Ras G12C inhibitors, namely AMG 510 [167] and MRTX849 [168]. The effect of these two inhibitors on tumor growth was assessed in 20 cell lines. Both the inhibitors showed mutant-selective inhibition of tumor growth with a marked effect on K-Ras (mutant-G12C)–ERK–MAPK and mTOR–S6K kinase pathways. In vivo studies in tumor-bearing mice fortified the anti-tumor effects of AMG 510 and MRTX849. Early clinical trials as reported by Amgen show the partial response of diminished tumor size in patients with non-small-cell lung cancer (NSCLC). Mirati Therapeutics also reported similar effects on the administration of MRTX489 to NSCLC, but the results in CRC patients in both cases were less promising. Although K-Ras signaling was attenuated in cell lines treated with MRTX849 and AMG510, the degree of sensitivity to these inhibitors varied between cell lines. A combinatorial therapy using AMG510 and a MEK inhibitor showed augmented efficacy of treatment. A major limitation of these two inhibitors is that neither AMG510 nor MRTX849 could affect PI3K signaling. PI3K is one important effector of Ras isoforms and it activates Akt family Ser/Thr kinases crucial for inhibition of apoptosis and cell survival [169]. Its importance in K-Ras signaling cannot be overlooked. To fully realize the potential of such K-Ras inhibitors additional combination strategies, as well as efficient targeting of most if not all signaling pathways downstream to K-Ras are to be addressed. As mentioned in the above sections compensatory or alternative mechanisms exist to compensate for the loss of a functional protein. This leads to acquired resistance to inhibitors. For instance– resistance against anaplastic lymphoma kinase (ALK) and EGFR inhibitors developed within 1–2 years of treatment because of mutations at drug binding sites and activation of alternative RTKs that continued downstream signaling [170]. Other K-Ras covalent inhibitors currently in a clinical trial include JNJ-74699157 (ARS-3248), LY3499446, etc. [171].

Sakamoto et al., obtained KRpep-2 (Ac-RRCPLYISYDPVCRR-NH2) through random screening of phage-displayed peptide libraries against K-Ras G12D. KR pep-2 selectively inhibited K-Ras G12D and significantly suppressed phosphorylation of ERK. A 30 μM concentration, KRpep-2 inhibited the proliferation of cancer cell line A427 as well [172]. Another approach used was to develop two recombinant peptides using amino acids 5-21 of K-Ras G12D (SP2) coupled to *Diptheria* toxin (DTT) as a carrier. Both these peptides DTT-SP4 and DTSP when administered as vaccines in combination with alum and cytosine phosphoguanine (CpG) gave rise to SP-specific humoral and cell-mediated immune response in CT26 mouse tumor models [173]. Tran et al. identified four CD8+ T cell clonotypes specific for K-Ras G12D from infiltrating lymphocyte population in metastasizing colorectal cancer. Adoptive transfer of these infiltrating lymphocytes leads to regression of lung metastasis in all 7 lung lesions. However, one lesion out of these 7 (lesion3) showed progression due to the loss of chromosome 6 haplotype that encodes for class I MHC molecule required for tumor recognition by K-Ras G12D-specific T cells [174]. Hence the adoptive transfer of tumor-infiltrating lymphocytes (TIL) offers a promising therapeutic approach against codon-specific mutation. The limitation of this approach lies in the fact that TIL only recognizes patient-specific antigens, and thus requires patient-specific T cells for each therapy. Moderna (Cambridge, MA, USA) and Merck (Darmstadt, Germany) are currently collaborating to test mRNAs vaccines against K-Ras tumors. mRNA act as a delivery vector to introduce tumor-specific antigens (TSA) and tumor-associated antigens (TAA) into cells. Once expressed and presented by MHCs, these antigens can mount both B Cell-mediated humoral as well as and CD4+/CD8+ cytotoxic immunity to clear malignant cells. A combinatorial therapy for pancreatic cancer using mRNA-5671 (encoding K-Ras neoantigen) with KEYTRUDA (a PD-1 specific inhibitor) is currently under clinical trial [175]. Although these studies open up the possibility of targeting G12D mutants, it yet remains in only a preliminary state of therapeutic plausibility.

K-Ras G12V mutants with a very low intrinsic GTPase activity account for about 30% of all Ras mutations in tumors. It remains active for a very long period [176]. Researchers have developed Proteolysis Targeting Chimeras (PROTAC)-like degradation-resistant monobody called 12VC1 that recognizes and noncovalently inhibited only active K-Ras mutants G12C and G12V [177]. Antibodies generally fail to detect mutant epitopes generated by cancer cells as they are present inside the cell. Single-chain variable fragments specific for these mutant peptides bound to HLA can be used to generate antibodies called mutation-associated neoantigen (MANAbodies). Previous studies have reported TCR-mimic antibody which targets K-Ras G12V/HLA-A*0201 [178]. This approach was further modified using antibody-drug conjugate (ADC) as TCRm-ADCs (2A5-MMAE and 2E8-MMAE) which showed higher specificity and lower toxicity [179]. Despite these signs of progress targeting K-Ras G12V remains a challenge unmet.

Different K-Ras G12 mutant subtypes differently induct downstream signaling molecule and thus differ in their sensitivity to specific drugs. For example, G12C phosphorylates ERK to a higher level than G12D which is supported by the finding that MEK inhibitor selumetinib is more effective in K-Ras G12C tumors when compared to G12D tumors [180,181]. Allele-specific mutation in K-Ras in different patient populations further adds complexity to finding an inhibitor and limits the monotherapeutic approach. Table 3 shows the various drugs/interventions currently in different phases of clinical trials for Ras isoforms. The biological differences due to the variability of K-Ras mutations are not well understood. The most common allele-specific mutation in K-Ras is K-Ras G12D and K-Ras-G12V. In pancreatic ductal adenocarcinoma (PDAC), G12D has low survivability whereas G12R has a higher probability of survival. In the case of non-small cell lung cancer (NSCLC), G12V or G12C mutant patients have a higher probability of survival when compared to other codon 12 mutations [182]. Also, K-Ras mutations co-occurring with mutations in tumor-suppressor genes may further lead to different biological behavior [183] and hence responsiveness to therapeutic agents. 

Comparative analysis of single and multiple mutations in mutant colorectal cancer (mCRC) shows K-Ras mutations in about 90% of patients. The most common mutation is present in exon 2 (92%), while 10% of the patients had multiple mutations. The median progression-free survival (PFS) and overall survival (O.S) was longer in patients with multiple mutations (22.7 months, 40.7 months) when compared to patients with single mutations (8.8 months, 12.8 months) [184] A detailed study on the clinical pathology and prognosis of the different K-Ras mutants shows that p.G12C and p.G12S variants are the most aggressive clinical subtypes in mCRC patients. A median follow-up performed 25.6 months post-diagnosis, shows the median survival was 7.3 months and 5 months in patients with K-Ras G12C and K-Ras G12S mutations respectively [185]. Patients with mutations in K-Ras exon 2 codons 12, 13 and N-Ras exon 2-4 are resistant to anti-EGFR treatment. The role of K-Ras in the negative prognosis of mCRC was observed in patients under different treatment regimens namely- irinotecan, oxaliplatin, and bevacizumab in various combinations. The lowest PFS, as well as OS, was observed in mCRC with K-Ras mutations. When compared to non-K-Ras mutated tumors, the PFS and OS of K-Ras G12C, K-Ras G13D were inferior. Although mutations in K-Ras G12V have a negative prognostic effect on PFS, K-Ras G12D and K-Ras G12V did not have any significant effect on OS [186]. In another study, the effect of K-Ras and N-Ras mutation on the prognosis of mCRC specifically in the case of liver-metastasis (LiM) and lung- metastasis (LuM) was assessed. K-Ras as well as N-Ras mutation affected the PFS and the OS of mCRC patients in the case of (LiM) but not in (LuM) [187]. In a subset of CRC patients who underwent hepatic resection, K-Ras mutations were negatively associated with OS and relapse-free survival (RFS) as well [188]. The differences in the percentage of K-Ras mutation as well as the exon specific mutation in different studies may be attributed to ethnic and environmental factors. 

The genetic dynamics in mCRC patients may play a decisive role in response to treatment. On analysis of the mutational trajectory of K-Ras in a cohort of CRC patients, two divergent subsets were identified. One subset consists of K-Ras mutation (mutK-Ras) in primary tumor to wild-type K-Ras (wtK-Ras) in metastatic form (8.8%), whereas the second subset consists of wtK-Ras in primary tumors to mutK-Ras in metastatic form (21.1%). This genetic heterogeneity could be classified as a progressive genetic trajectory with patients having lower survival, poor responsiveness to chemotherapy (CT), and a regressive mutational trajectory, which shows higher survival and high responsiveness to chemotherapy. As mentioned above, the primary regions of metastasis in CRC are the lungs and liver. However, in the case of tumor perorations, peritoneal carcinomatosis may also occur leading to the oligometastatic condition. A regressive mutational trajectory with loss of K-Ras mutation during metastasis is associated with oligometastatic status in mCRC. Study shows that capecitabine and oxaliplatin-based adjuvant chemotherapy preceded the evolution from wtK-Ras to mutK-Ras whereas a regressive mutational trajectory (mutK-Ras to wtK-Ras) was observed as a response to CT or CT along with bevacizumab. These observations provide evidence for chemotherapy-induced genetic remodeling. 

The genetic heterogeneity in the abovementioned subsets also affected the OS. In the case of the subsets wtK-Ras (tumor) to wtK-Ras (metastasis) and mutK-Ras (tumor) to mutK-Ras (metastasis), the median OS was 27.5 and 9.6 months respectively. Whereas for wtK-Ras (tumor) to mutK-Ras (metastasis) the median OS was 12.1 months and for mutK-Ras (tumor) to wtK-Ras (metastasis) the median OS was not reached [189]. In the case of lung-specific oligometastatic CRC, analysis of primary and metastatic tumors reveals a decrease in the tumor mutation burden (TMB), regressive mutations in K-Ras, and SMAD4, and scarce T cell infiltrate. A point mutation in ERBB2 (a member of tyrosine kinase receptor) which is involved amplification of cancers was also observed [190]. Using a model of CRC, Ottaiano et al. also showed that the mutational direction of oligometastatic and polymetastatic disease is different. Oligometastatic status is characterized by the loss of K-Ras and SMAD4 mutations (regressive mutational evolution), whereas a progressive mutational evolution with gain in K-Ras, SMAD4, B-Raf, and PI3KCA characterizes polymetastatic status. Patients with back mutations in K-Ras and SMAD4 showed higher infiltration of granzyme B+ (GrzB+) T cells in the metastatic tumor microenvironment. Conversely, patients with forward mutations showed a lower infiltration of GrzB+ T cells [191]. This forward mutation also affects the prognosis, e.g., K-Ras and PI3KCA mutation is associated with resistance to anti-cancer drugs and aggressive CRC phenotype [192]. A better understanding of such characteristics of codon-specific mutants, co-occurring mutations, genetic heterogeneity, and functionally distinct allelic forms is a prerequisite to generating efficient inhibitors

Bacterial protein toxins and effector proteins can specifically target heterotrimeric G Proteins or small GTPases of the Rho and Ras family to modify host cell signaling [193]. While the vast majority of toxins modify Rho GTPases, there are also examples of Ras being the target. To date, however, there is no known bacterial toxin that specifically modifies only Ras. Toxins that act on Ras include TcsL and TcsH from *Clostridium sordellii* (UDP glycosylation), TpeL from *Clostridium perfringens* (*N*-acetylglucosamination), ExoS from *Pseudomonas aeruginosa* (ADP ribosylation), and DUF5Vv (endopeptidase) from *Vibrio vulnificans*. 

The lethal toxin of *C. sordellii*, TcsL, modifies H, K, and N-Ras at Thr35 in the switch I region by catalyzing the transfer of glucose from UDP-Glucose to the threonine residue [194]. This keeps the protein in an inactive, GDP-bound state which eventually causes cell death through cell cycle arrest and induction of apoptosis [195,196]. Large cytotoxin TpeL from *C. perfringens* catalyzes *N*-acetylglucosamination of the same Thr35 residue in all main types of oncogenic Ras. Again, the toxin modification resulted in a loss of MapK activity and cell death [197]. *Pseudomonas* ExoS is an effector protein that is transferred into host cells via a type III secretion system. Among many other cellular targets, ExoS inhibits Ras through ADP ribosylation of the residues Arg41 and Arg128, both outside the switch I region. Still, this modification attenuates loading with GTP and blocks interaction with downstream effectors [198]. Last but not least, a new family of “Ras/Rap specific proteases” (RRSP) was recently defined that comprises toxins from *Vibrio vulnificus*, *Aeromonas hydrophila,* and the insect-specific pathogen *Photorhabdus asymbiotica* [199]. DUF5_Vv_ belongs to the class of multifunctional-autoprocessing repeats-in-toxin (MARTX) toxins. The prototypic DUF5_Vv_ of *Vibrio vulnificus* targets Ras and Rap located at the plasma membrane with its endopeptidase activity which cleaves the GTPase between Y32 and D33 and prepares it for subsequent protein degradation [196]. MARTX toxins translocate across the plasma membrane to deliver their effector proteins from the holotoxin through autoprocessing [200]. DUF5_Vv_ targets all isoforms of Ras as well as the most important oncogenic mutants which results in cell death by inhibition of cell division. Thus, this toxin might be employed to specifically delete Ras and kill tumor cells. Indeed, such an approach was later employed by Schorch et al., where the catalytically active domain of TpeL was coupled to the binding domain of the anthrax toxin binding and translocation component PA (protective antigen) [197]. To increase the specificity for tumor cells, a CD46 interaction domain was engineered into the binding region, as CD46 is often overexpressed on cancer cells. In this in vitro study, human pancreatic cancer Capan-2 cells were shown to get successfully deleted. 

Such immunotoxins are elegant tools to specifically target and destroy cancer cells and several immunotoxins are in clinical or preclinical evaluation [201]. Three immunotoxins have already been approved by the FDA. The cell death-inducing diphtheria toxin (DT) can be coupled to the cytokines IL-2 or IL-3 (DT-IL3, DT-IL3), respectively, and cells that express the cognate receptor take up the toxin and die due to the inhibition of protein translation by DT [202]. Lumoxiti makes use of the *Pseudomonas aeruginosa* exotoxin A coupled to an anti-CD22 antibody that recognizes the phosphoglycoprotein CD22 expressed on B cells. However, some restrictions prevent the easy application of immunotoxins. Depending on the receptor that is recognized by the binding domain, the toxin might also get delivered into healthy cells causing equally efficient, but unwanted cell death. Additionally, protein-derived drugs will be recognized by the adaptive immune system through MHC antigen presentation, resulting in the production of protective antibodies. Zahaf and Schmidt also rightly pointed out that solid tumors are of heterogeneous nature so that immunotoxins would only target parts of the tumor, making it necessary to use immunotoxins in combination with conventional chemotherapeutics [201].

From the above discussion, it is clear that the ‘one size fits all’ approach cannot be adopted when designing inhibitors against Ras isoforms. From the localization in the plasma membrane and different organelle to their behavior on activation by the same or different receptors, multiple strata of functional complexity exist. In addition to this, the functioning of Ras in an oncogenic setup versus a normal cell is quite different. Our understanding of the effect of autonomous or synergistic/antagonistic signaling arising from various cellular compartments (mitochondria, Golgi, E.R, endosome, and nucleus) in deciding the signaling outcome is very limited. As mentioned in Section 5.5, few studies have shown activation of Ras isoforms by specific GEFs in these cellular compartments. However, a consolidated study of the distribution and activation of the RasGEFs–Ras isoforms–Ras effector’s axis upon receptor stimulation is yet to be elucidated. Using SOS1 and Ras GRF1, Tian et al. have identified in the catalytic domain of GEF, a 52 aa (amino acid) segment that forms helices B and C as the key determinant of signaling specificity. On replacement of amino acids in SOS1 with analogous amino acids from Ras GRF1, SOS1 could activate the target GTPase of Ras GRF1 i.e., R-Ras. However, on replacement of Ras GRF1 sequences with that of SOS1, Ras GRF1 failed to activate R-Ras and H-Ras. This suggests that different Ras GEFs have different mechanisms to activate target GTPase [203]. Studies that show amino acid sequence specificity in Ras GEFs and their corresponding Ras isoforms targets under different signaling contexts would enable researchers a better understanding of divergence of signaling. Studies are pointing out possible new functions of Ras isoforms. RanGTPase is known to be a key regulator of nucleocytoplasmic transport of cargoes in and out of the nucleus. With studies showing the presence of H-Ras in the nucleus, many questions arise as to the function of Ras in the nucleus, regarding its mechanism of activation, nuclear proteins that may bind to H-Ras, and the signaling pathway. Whether H-Ras performs a similar function as RanGTPase remains to be elucidated. Most studies are performed with oncogenic Ras mutants, however, a detailed assessment of the functioning of these isoforms in a normal cell is equally important. With studies pointing out novel structural differences in H, K, and N-Ras, as well as the implications of these structural differences in its activation, the need to collate all these findings in a comprehensible form is more than ever. The functional specificity and context-dependent signaling are to be taken into consideration while designing an inhibitor/drug to treat various Ras-dependent pathologies or “rasopathies”.

## Figures and Tables

**Table 1:** The table has been compiled using data from COSMIC v92 (Catalog Of Somatic Mutations In Cancer by Sangers Institute (https://cancer.sanger.ac.uk/cosmic, accessed on 13 June 2021)).

S. No.	Primary Tissue Type	(Mutated) Total Samples Tested	% of Point Mutations	(Mutated) Total Samples Tested	% of Point Mutations	(Mutated) Total Samples Tested	% of Point Mutations
		H-Ras	H-Ras	K-Ras	K-Ras	N-Ras	N-Ras
1	Adrenal gland	(45)1303	3.45	(1)1331	0.88	(13)1276	1.02
2	Autonomic ganglia	(6)1597	0.38	(1)1572	0.06	(11)1673	0.66
3	Biliary tract	(9)1728	0.52	(958)5367	19.49	(58)2133	2.72
4	Bone	(8)1060	0.75	(14)1184	1.27	(13)1209	1.08
5	Breast	(54)8192	0.66	(150)11297	1.42	(33)7763	0.43
6	Central nervous system	(11)4150	0.27	(47)4881	1.21	(54)4800	1.13
7	Cervix	(28)1249	2.24	(151)2569	6.03	(10)1191	0.84
8	Endometrium	(14)2384	0.59	(720)4663	15.89	(53)1637	3.24
9	Eye	340	−	(7)476	1.47	(40)888	4.5
10	Fallopian tube	3	−	9	−	6	−
11	Female genital tract (site indeterminate)	20	−	(1)24	8.33	22	−
12	Gastrointestinal tract (site indeterminate)	1	−	(70)1083	6.46	477	−
13	Genital tract	(2)285	0.7	(36)576	6.42	(12)565	2.12
14	Haematopoietic and lymphoid	(24)12623	0.19	(1124)24788	4.91	(2517)28829	8.73
15	Kidney	(7)31127	0.22	(41)4277	1.01	(12)3345	0.36
16	Large intestine	(56)5989	0.94	(25902)80015	33.4	(617)16211	3.81
17	Liver	(7)2902	0.24	(112(3604	3.3	(34)3164	1.07
18	Lung	(45)7450	0.6	(6419)43284	16.08	(145)16486	0.88
19	Mediastinum	1	−	1	−	1	−
20	Meninges	(12)283	4.24	(4)317	1.26	(23)359	6.41
21	NS	(17)1251	1.36	(103)1808	6.19	(458)2674	17.13
22	Oesophagus	(9)2668	0.34	(71)3203	2.28	(5)2142	0.23
23	Ovary	(4)1677	0.24	(899)6913	13.57	(31)1916	1.62
24	Pancreas	(3)3135	0.1	(6767)12289	56.38	(26)3513	0.74
25	Paratesticular tissues	*	*	1	−	*	*
26	Parathyroid	(2)151	1.32	136	−	135	−
27	Penis	(25)209	11.6	(13)433	3.23	(3)128	2.34
28	Pericardium		−	3	−	2	−
29	Perineum		−	2	−	2	−
30	Peritoneum		−	(166)371	45.28	(1)149	0.67
31	Pituitary	(12)364	3.3	389		390	
32	Placenta		−	14	−	6	−
33	Pleura		−	(7)739	0.95	(4)581	0.69
34	Prostate	(61)3978	1.53	(136)4827	2.86	(30)3953	0.76
35	Salivary gland	(93)783	11.88	(12)701	1.71	(5)573	0.87
36	Skin	(648)6529	9.92	(177)6286	3.07	(2408)15500	15.54
37	Small intestine	(1)316	0.32	(256)1232	21.27	(2)420	0.48
38	Soft tissue	(74)2767	2.67	(118)4577	2.67	(70)3171	2.21
39	Stomach	(37)3399	1.09	(389)6472	6.13	(28)2597	1.08
40	Testis	(5)675	0.74	(61)1199	6.51	(26)1041	2.5
41	Thymus	(9)393	2.29	(9)652	1.38	(4)469	0.85
42	Thyroid	(451)10503	4.29	(239)12387	2.11	(933)11422	8.17
43	Upper aerodigestive tract	(228)3911	5.83	(95)5146	2.06	(51)3449	1.48
44	Urinary tract	(321)3727	8.61	(155)2803	5.71	(43)2514	1.71
45	Vagina		−	5	−	4	−
46	Vulva	(20)175	11.43	(2)199	1.01	175	−

− No mutation; * No data.

**Table 2:** Genetic heterogeneity in Noonan syndrome with the name of the mutated gene in each subtype. The table is compiled using data from OMIM- Online Mendelian Inheritance in Man^®^-https://www.omim.org/ (Online Mendelian Inheritance in Man, OMIM^®^. McKusick-Nathans Institute of Genetic Medicine, Johns Hopkins University (Baltimore, MD), World Wide Web URL: https://omim.org/) (accessed on 30 May 2021).

S. No.	Subtype of NS	Mutated Gene
1	NS1	PTPN11
2	NS2	LZTR1
3	NS3	KRAS
4	NS4	SOS1
5	NS5	RAF1
6	NS6	NRAS
7	NS7	BRAF
8	NS8	RIT1
9	NS9	SOS2
10	NS10	LZTR1
11	NS11	MRAS
12	NS12	RRAS2
13	NS13	MAPK1

**Table 3:** Various drug/interventions currently under clinical trial for H-Ras, K-Ras, and N-Ras. The table is compiled using data from NIH-U.S library of medicine- ClinicalTrials.gov (www.clinicaltrials.gov), (accessed on 26 May 2021).

S. No.	Category	Condition	Intervention/Treatment	Clinical trial status
1	H-Ras mutation	HRAS Gene Mutation HNSCC	Drug: Tipifarnib Device: H-Ras Detection Assay	Phase 2
2	H-Ras mutation	Thyroid Cancer Squamous Cell Carcinoma Head and Neck Cancer (HNSCC) H-Ras Mutant Tumor Other Squamous Cell Carcinoma (SCC) With H-Ras Mutant Tumor	Drug: Tipifarnib	Phase 2
3	H-Ras mutation	Recurrent Adrenal Gland Pheochromocytoma Recurrent Ectomesenchymoma Recurrent Ependymoma Recurrent Ewing Sarcoma Recurrent Hepatoblastoma Recurrent Kidney Wilms Tumor Recurrent Langerhans Cell Histiocytosis Recurrent Malignant Germ Cell Tumor Recurrent Malignant Glioma Recurrent Medulloblastoma Recurrent Melanoma Recurrent Neuroblastoma Recurrent Non-Hodgkin Lymphoma Recurrent Osteosarcoma Recurrent Peripheral Primitive Neuroectodermal Tumor Recurrent Rhabdoid Tumor Recurrent Rhabdoid Tumor of the Kidney Recurrent Rhabdomyosarcoma Recurrent Soft Tissue Sarcoma Recurrent Thyroid Gland Carcinoma Recurrent WHO Grade II Glioma Refractory Adrenal Gland Pheochromocytoma Refractory Ependymoma Refractory Ewing Sarcoma Refractory Hepatoblastoma Refractory Langerhans Cell Histiocytosis Refractory Malignant Germ Cell Tumor Refractory Malignant Glioma Refractory Medulloblastoma Refractory Melanoma Refractory Neuroblastoma Refractory Non-Hodgkin Lymphoma Refractory Osteosarcoma Refractory Peripheral Primitive Neuroectodermal Tumor Refractory Rhabdoid Tumor Refractory Rhabdoid Tumor of the Kidney Refractory Rhabdomyosarcoma Refractory Soft Tissue Sarcoma Refractory Thyroid Gland Carcinoma Refractory WHO Grade II Glioma	Drug: Tipifarnib	Phase 2
4	H-Ras mutation	Non-Small Cell Lung Cancer	Drug: Tipifarnib	Phase 2
5	H-Ras mutation	Urothelial Carcinoma	Drug: Tipifarnib	Phase 2
6	H-Ras mutation	Colorectal Cancer	Drug: ISIS 2503	Phase 2
7	H-Ras mutation	Pancreatic Cancer	Drug: ISIS 2503	Phase 2
8	H-Ras mutation	Cancer Malignancy Neoplasia Neoplasm Neoplasm Metastasis Colon Cancer Colonic Neoplasms Colon Cancer Liver Metastasis Metastatic Cancer Metastatic Melanoma Metastatic Colon Cancer Metastatic Lung Cancer Non-Small Cell Lung Cancer Metastatic Pancreatic Cancer Pancreas Cancer Pancreas Adenocarcinoma Pancreas Neoplasm Metastatic Nonsmall Cell Lung Cancer Metastatic Pancreatic Cancer	Drug: ASN007: ascending doses Drug: ASN007 RD	Phase 1
1	K-RasG12C	Neoplasms Advanced Solid Tumors Non-small Cell Lung Cancer Colorectal Cancer	Drug: JNJ-74699157	Phase 1
2	K-RasG12C	Advanced Cancer Metastatic Cancer Malignant Neoplastic Disease	Drug: MRTX849 Drug: TNO155	Phase ½
3	K-RasG12C	Advanced Non-Small Cell Lung Cancer Metastatic Cancer	Drug: MRTX849 in Combination with Pembrolizumab	Phase 2
4	K-RasG12C	Advanced Cancer Metastatic Cancer Malignant Neoplastic Disease	Drug: MRTX849 Drug: Pembrolizumab Drug: Cetuximab Drug: Afatinib	Phase 1/2
5	K-RasG12C	Metastatic Non-Small Cell Lung Cancer Advanced Non-Small Cell Lung Cancer	Drug: MRTX849 Drug: Docetaxel	Phase 3
6	K-RasG12C	Advanced Colorectal Cancer Metastatic Colorectal Cancer	Drug: MRTX849 Biological: Cetuximab Drug: mFOLFOX6 Regimen Drug: FOLFIRI Regimen	Phase 3
7	K-RasG12C	Non-Small Cell Lung Cancer Colorectal Cancer Advanced Solid Tumors	Drug: GDC-6036 Drug: Atezolizumab Drug: Cetuximab Drug: Bevacizumab Drug: Erlotinib	Phase 1
8	K-RasG12C	Lung Adenocarcinoma Lung Non-Small Cell Carcinoma Recurrent Lung Non-Squamous Non-Small Cell Carcinoma Stage IV Lung Cancer AJCC v8 Stage IVA Lung Cancer AJCC v8 Stage IVB Lung Cancer AJCC v8	Drug: Sotorasib	Phase 2
9	K-RasG12C	KRAS G12C Mutant Solid Tumors Carcinoma, Non-Small-Cell Lung Carcinoma, Colorectal Cancer of Lung Cancer of the Lung Lung Cancer Neoplasms, Lung Neoplasms, Pulmonary Pulmonary Cancer Pulmonary Neoplasms	Drug: JDQ443 Drug: TNO155 Biological: spartalizumab	
10	K-RasG12C	Advanced/Metastatic Solid Tumors With KRAS p.G12C Mutation	Drug: AMG 510	Phase 1
11	K-RasG12C	Advanced Solid Tumors Kirsten Rat Sarcoma (KRAS) pG12C Mutation	Drug: Sotorasib Drug: PD1 inhibitor Drug: MEK inhibitor Drug: SHP2 allosteric inhibitor Drug: Pan-ErbB tyrosine kinase inhibitor Drug: PD-L1 inhibitor Drug: EGFR inhibitor Drug: Chemotherapeutic regimen Drug: PD-1 inhibitor Drug: mTOR inhibitor Drug: CDK inhibitor Drug: VEGF inhibitor	Phase 1
12	K-RasG12C	KRAS p.G12C Mutant Advanced Solid Tumors	Drug: AMG 510 Drug: Anti PD-1/L1 Drug: Midazolam	Phase 1/2
13	K-RasG12C	Non-Small-cell Lung Cancer Locally Advanced Unresectable NSCLC Locally Advanced Metastatic NSCLC	Drug: AMG 510	
14	K-RasG12C	KRAS p, G12c Mutated /Advanced Metastatic NSCLC	Drug: AMG 510 Drug: Docetaxel	Phase 3
15	K-RasG12C	Solid Tumor, Adult NSCLC CRC	Drug: D-1553 Drug: Other	Phase 1/2
16	K-RasG12C	Advanced EGFR mutant Non-Small cell Lung Cancer (NSCLC), KRAS G12-mutant NSCLC, Esophageal Squamous Cell Cancer (SCC), Head/Neck SCC, Melanoma	Drug: TNO155 Drug: TNO155 in combination with EGF816 (nazartinib)	Phase 1
17	K-RasG12C/ K-RasG12D	Advanced or Metastatic Solid Tumors	Drug: TAS0612	Phase 1
18	K-RasG12C	Tumor, Solid	Drug: BBP-398 (Formerly Known as IACS-15509)	Phase 1
19	K-RasG12C	Carcinoma, Non-Small Cell Lung	Drug: carboplatin Drug: paclitaxel Drug: Bevacizumab Drug: Pemetrexed Drug: cisplatin	Phase 3
20	K-RasG12C	KRAS Gene Mutation Recurrent Lung Non-Small Cell Carcinoma Stage IV Lung Non-Small Cell Cancer AJCC v7	Drug: Docetaxel Other: Laboratory Biomarker Analysis Drug: Trametinib	Phase 2
21	K-RasG12C	Advanced Solid Tumor Non-Small Cell Lung Cancer Colorectal Cancer	Drug: LY3499446 Drug: Abemaciclib Drug: Cetuximab Drug: Erlotinib Drug: Docetaxel	Phase 1/2
1	K-RasG12D	Non-Small Cell Lung Cancer	Drug: Bortezomib Drug: Acyclovir	Phase 2
2	K-RasG12D	Minimal Residual Disease KRAS G12D KRAS G12R NRAS G12D NRAS G12R Pancreatic Ductal Adenocarcinoma Colorectal Cancer Non-small Cell Lung Cancer Ovarian Cancer Cholangiocarcinoma Bile Duct Cancer Gallbladder Carcinoma	Drug: ELI-002 (Dose Escalation) Drug: ELI-002 (at the RP2D) Other: Observation	Phase 1/2
3	K-RASG12D	TNBC—Triple-Negative Breast Cancer Head and Neck Squamous Cell Carcinoma Squamous Cell Carcinoma of Anal Canal Uveal Melanoma Glioblastoma Colorectal Cancer Chordoma Squamous Cell Carcinoma of the Lung KRAS G12D KRAS G13D EGFR Amplification Epithelial Ovarian Cancer Hepatocellular Carcinoma Anaplastic Thyroid Cancer Pancreas Cancer	Drug: BCA101 Drug: Pembrolizumab	Phase 1
4	K-RasG12D	Advanced or Metastatic Solid Tumors	Drug: TAS0612	Phase 1
1	K-RasG12V	Pancreatic Cancer Pancreatic Neoplasms Pancreatic Ductal Adenocarcinoma Advanced Cancer	Drug: Cyclophosphamide Drug: Fludarabine Biological: Mutant KRAS G12V-specific TCR transduced autologous T cells Drug: Anti-PD-1 monoclonal antibody	Phase 1/2
2	K-RasG12V	Non-Small Cell Lung Cancer KRAS Activating Mutation	Drug: VS-6766 Drug: VS-6766 and Defactinib	Phase 2
1	K-RasG13D	TNBC—Triple-Negative Breast Cancer Head and Neck Squamous Cell Carcinoma Squamous Cell Carcinoma of Anal Canal Uveal Melanoma Glioblastoma Colorectal Cancer Chordoma Squamous Cell Carcinoma of the Lung KRAS G12D KRAS G13D EGFR Amplification Epithelial Ovarian Cancer Hepatocellular Carcinoma Anaplastic Thyroid Cancer Pancreas Cancer	Drug: BCA101 Drug: Pembrolizumab	Phase 1
1	N-Ras mutation	KRAS Gene Mutation Metastatic Colorectal Carcinoma NRAS Gene Mutation Stage III Colorectal Cancer AJCC v8 Stage IIIA Colorectal Neuroendocrine Tumor AJCC v8 Stage IIIB Colorectal Cancer AJCC v8 Stage IIIC Colorectal Cancer AJCC v8 Stage IV Colorectal Cancer AJCC v8 Stage IVA Colorectal Cancer AJCC v8 Stage IVB Colorectal Cancer AJCC v8 Stage IVC Colorectal Cancer AJCC v8 Unresectable Carcinoma	Drug: Binimetinib Drug: Palbociclib Drug: Trifluridine and Tipiracil Hydrochloride	Phase 2
2	N-Ras mutation	BRAF V600E Negative KRAS Gene Mutation Negative Locally Advanced Unresectable Colorectal Adenocarcinoma Metastatic Colorectal Adenocarcinoma NRAS Gene Mutation Negative Stage III Colorectal Cancer AJCC v8 Stage IIIA Colorectal Cancer AJCC v8 Stage IIIB Colorectal Cancer AJCC v8 Stage IIIC Colorectal Cancer AJCC v8 Stage IV Colorectal Cancer AJCC v8 Stage IVA Colorectal Cancer AJCC v8 Stage IVB Colorectal Cancer AJCC v8 Stage IVC Colorectal Cancer AJCC v8	Biological: Cetuximab Drug: Irinotecan Biological: Panitumumab Drug: Regorafenib	Phase 2
3	N-Ras mutation	Solid Tumor	Drug: ARQ 736	Phase 1
4	N-Ras mutation	Cancer Lung Cancer Metastatic Immunotherapy	Drug: PDR001	Phase 2
5	N-Ras mutation	Metastatic or Unresectable Cutaneous Melanoma	Drug: MEK162 Drug: Dacarbazine	Phase 3
6	N-Ras mutation	BRAF or NRAS Mutant Metastatic Melanoma	Drug: MEK162	Phase 2
7	N-Ras mutation	Advanced Lymphoma Advanced Malignant Solid Neoplasm Hematopoietic and Lymphoid Cell Neoplasm Refractory Lymphoma Refractory Malignant Solid Neoplasm Refractory Plasma Cell Myeloma	Drug: Binimetinib	Phase 2
8	N-Ras mutation	Solid Tumor	Drug: HM95573	Phase 1
9	N-Ras mutation	Lung Cancer, Non-Small Cell	Drug: GSK1120212 Drug: docetaxel	Phase 2
10	N-Ras mutation	Metastatic Malignant Solid Neoplasm Refractory Malignant Solid Neoplasm Unresectable Malignant Solid Neoplasm	Biological: Navitoclax Drug: Trametinib	Phase 1/2
